# Highly Efficient Capture of Heavy Metal Ions on Amine-Functionalized Porous Polymer Gels

**DOI:** 10.3390/gels9040297

**Published:** 2023-04-02

**Authors:** Xue He, Jumu Xia, Jieli He, Kezhen Qi, Anzhong Peng, Yong Liu

**Affiliations:** College of Pharmacy, Dali University, Dali 671003, China

**Keywords:** porous polymer gels, post-synthetic modification, adsorption sites, super-high Pb^2+^ uptake, environmental remediation

## Abstract

Porous polymer gels (PPGs) are characterized by inherent porosity, a predictable structure, and tunable functionality, which makes them promising for the heavy metal ion trap in environmental remediation. However, their real-world application is obstructed by the balance between performance and economy in material preparation. Development of an efficient and cost-effective approach to produce PPGs with task-specific functionality remains a significant challenge. Here, a two-step strategy to fabricate amine-enriched PPGs, NUT-21-TETA (NUT means Nanjing Tech University, TETA indicates triethylenetetramine), is reported for the first time. The NUT-21-TETA was synthesized through a simple nucleophilic substitution using two readily available and low-cost monomers, mesitylene and α, α′-dichloro-p-xylene, followed by the successful post-synthetic amine functionalization. The obtained NUT-21-TETA demonstrates an extremely high Pb^2+^ capacity from aqueous solution. The maximum Pb^2+^ capacity, q_m_, assessed by the Langmuir model was as high as 1211 mg/g, which is much higher than most benchmark adsorbents including ZIF-8 (1120 mg/g), FGO (842 mg/g), 732-CR resin (397 mg/g), Zeolite 13X (541 mg/g), and AC (58 mg/g). The NUT-21-TETA can be regenerated easily and recycled five times without a noticeable decrease of adsorption capacity. The excellent Pb^2+^ uptake and perfect reusability, in combination with a low synthesis cost, gives the NUT-21-TETA a strong potential for heavy metal ion removal.

## 1. Introduction

Environmental pollution has increasingly become a global issue, and the separation of natural and anthropogenic harmful pollutants in ecosystems is urgently needed, especially heavy-metal species [1,2,3]. Heavy metal ions are viewed as a worldwide danger to human health and the natural environment due to their high toxicity, difficult degradation, and bioaccumulation properties [4,5]. Nowadays, different strategies including adsorption separation, ion exchange [6], solvent extraction [7], chemical precipitation [8], nanofiltration [9], and reverse osmosis [10,11] have been proposed extensively for the remediation of toxic metal emissions [12,13,14,15]. Among them, the adsorption technique holds fascinating advantages including a low investment cost, mild operating conditions, and a high energy efficiency [16,17,18,19]. It has been proven that the high-performance adsorbents are the key to an efficient adsorption process, and the abundant surface porosity and active sites are two crucial factors [20,21].

Porous polymer gels (PPGs) have emerged as one of the most studied materials in terms of adsorption, separation, catalysis, semiconduction, and much more [22,23,24]. The structural diversity, tunable pore size, tailorable surface properties, and physicochemical stability make the PPGs a highly promising alternative for relieving environmental issues resulting from toxic heavy metals [25,26,27]. Amino groups can act as effective metal ion-philic adsorption sites to enhance the removal performance from wastewater run-off, and significant concerns have been reported [28,29,30]. For instance, recently, a series of triptycene-derived polymers were reported for heavy metal ion capture and the adsorption capacity of heavy metal ions depended on the suitable porosity, functional site density, and coordination modes. Also, the complex monomers, high-priced catalysts, and tedious reaction steps were inevitably involved, which made their practical application difficult to scale up [17]. Furthermore, various porous materials including porous silica, graphene oxide, and zeolites have been employed to remove the toxic metal ions for environmental remediation [31,32,33,34]. Many efforts have been committed to metal ion removal, while the development of a simultaneously efficient and economical approach to produce high-performance adsorbents remains a great challenge for materials engineering.

In this work, our main objectives are to investigate the relationship between the material structure and separation performance, and then to provide a reasonable strategy for the design of high-performance PPGs adsorbents. We successfully synthesized a new N-enriched polymer gel, denoted as NUT-21-TETA (NUT, Nanjing Tech University; TETA, triethylenetetramine), under mild conditions through a two-step method. The NUT-21-TETA was fabricated by a Friedel–Crafts alkylation reaction using the readily available and low-cost monomers (mesitylene, α, α′-dichloro-p-xylene, and triethylenetetramine). The experimental results demonstrate that the amino groups incorporated can adjust the pore structure due to their steric hindrance, and they work as the adsorption sites. The synergistic effect between the modified structure and the accessible sites can give rise to a greatly improved capture performance. The NUT-21-TETA thus possesses a much higher uptake (Pb^2+^, up to 1211 mg/g) than that of its counterpart, the NUT-21 without post-synthetic functionalization (Pb^2+^, 39 mg/g), and most other adsorbents reported to date. Furthermore, the removal of different heavy metal ions including Ni^2+^, Cu^2+^, Zn^2+^, Cr^3+^, and Fe^3+^ were also studied, which we expected to demonstrate an efficient and cost-effective strategy for designing the task-specific functional materials.

## 2. Results and Discussion

### 2.1. Structural and Surface Properties

**Figure 1:**
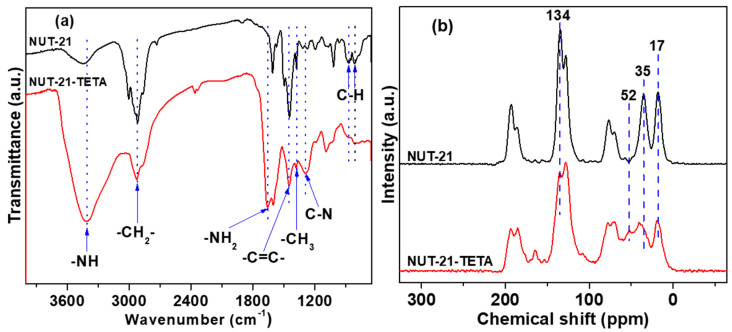
(**a**) IR spectra of NUT-21 and NUT-21-TETA, (**b**) solid-state ^13^ C NMR spectra of NUT-21 and NUT-21-TETA.

As shown in Figure 1a, the IR spectra of the NUT-21 and NUT-21-TETA exhibited intense peaks at 2920, 1450, and 1380 cm^−1^, which were assigned to the –CH_2_– stretching vibrations, C=C bending vibrations of the aromatic ring, and –CH_3_ stretching vibrations, respectively [35]. For the the NUT-21, the peaks at 868 and 813 cm^−1^ were ascribed to the C-H deformation vibration of ring hydrogens. The new and strong characteristic peaks at around 3380, 1650, and 1290 cm^−1^ appeared in the spectrum of the NUT-21-TETA, which were ascribed to the -NH stretching vibrations, –NH_2_ bending vibration, and C-N stretching vibration [36], indicating the existence of free-standing amine groups in the NUT-21-TETA. As depicted in Figure 1b, the solid state ^13^C-NMR spectra of the NUT-21 and NUT-21-TETA displayed strong peaks at 134, 35, and 17 ppm, assigned to the sp^2^ C of the aromatic rings, the methylene, and the methyl carbons, respectively [37,38]. For the NUT-21-TETA, the new characteristic peak at 52 ppm was originated from the carbon atoms connected to N atoms [37], indicating the creation of the post-synthetic functionalization again. The above experimental facts clearly suggest the successful fabrication of the NUT-21-TETA via a post-synthetic, amine-functionalized strategy under the given conditions. The NUT-21-TETA shows an amorphous characteristic based on its XRD patterns (Figure S1), displaying only a broad peak.

**Figure 2:**
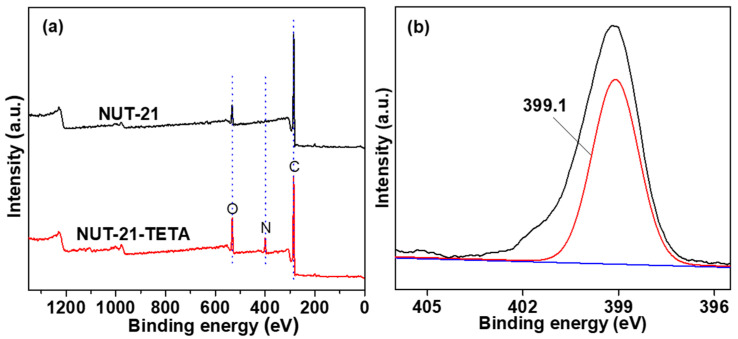
(**a**) XPS wide spectra of the NUT-21 and NUT-21-TETA, (**b**) XPS N 1s spectra of the NUT-21-TETA.

The elemental analysis for the C, H, and N contents of different samples was listed in Table 1. To investigate the modification degree based on the N content of the NUT-21-TETA, we inferred that about 46.2% of the methyl in the NUT-21 was ammoniated successfully. As can be observed from the XPS measurement (wide spectra) of the samples (Figure 2a), the N content of the NUT-21-TETA was consistent with the elemental measurement results. The status of N on the NUT-21-TETA was further explored by the XPS spectra (Figure 2b). The spectrum showed a peak at around 399.1 eV, which was ascribed to the post-modification amide nitrogen [39], providing further evidence for the successful introduction of amine groups that can work as active sites for heavy metal ion removal.

**Figure 3:**
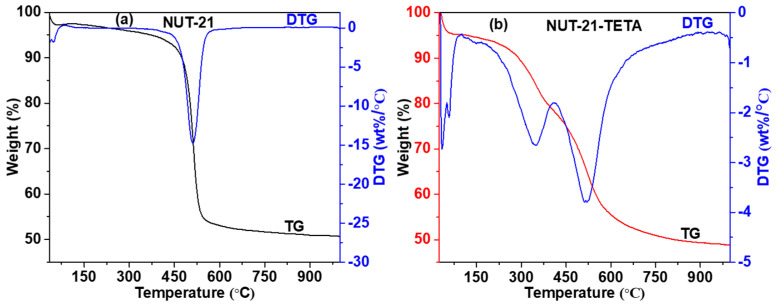
TG and DTG curves of (**a**) NUT-21, (**b**) NUT-21-TETA.

The thermal stability of samples was examined by TG and DTG measurement in an inert gas environment. As given in Figure 3, both the NUT-21-TETA and the counterpart NUT-21 showed a mass loss stage near 100 °C, which was ascribed to the desorption of some of the adsorbed water. Furthermore, the NUT-21-TETA displayed a higher mass loss than that of NUT-21 because the hygroscopic property of the NUT-21-TETA was improved after the incorporation of hydrophilic groups of –NH_2_. The mass loss stage near 500 °C can be ascribed to the collapse of the organic frameworks. The TG and DTG measurement results indicate that the samples prepared possess a high thermal stability. In the case of the NUT-21-TETA, a new characteristic mass loss stage at about 350 °C appeared, which was assigned to the dissociation of the grafted amino groups, confirming that the successful fabrication of the post-synthetically modified NUT-21-TETA was achieved.

**Figure 4:**
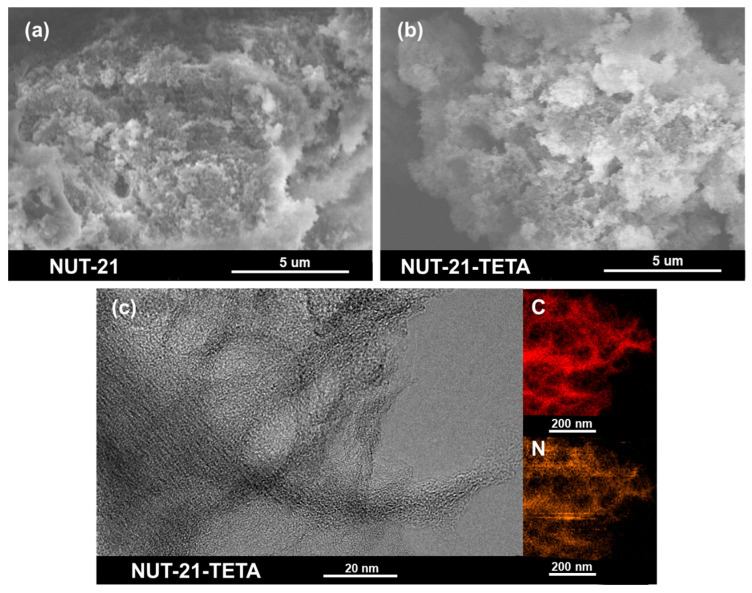
SEM images of (**a**) NUT-21, (**b**) NUT-21-TETA, and (**c**) TEM EDX mapping of the NUT-21-TETA.

The SEM measurement was employed to examine the surface morphologies of the NUT-21 and NUT-21-TETA. As displayed in Figure 4, the sponge-like structures with interconnected microparticles were observed on both samples. The TEM images (Figure 4c) presented the structural character of wormhole-like micropores in the NUT-21-TETA. Furthermore, the N element was uniformly dispersed on the NUT-21-TETA, as evidenced by the elemental mapping.

**Figure 5:**
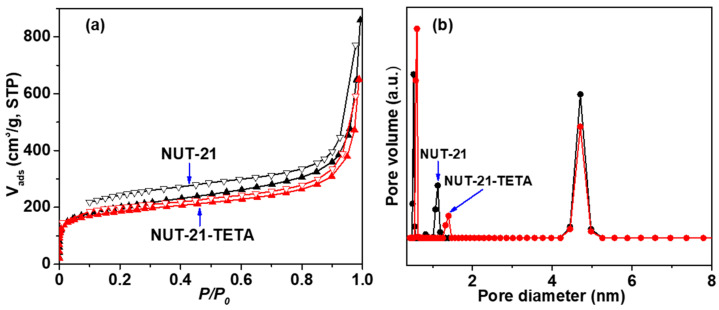
(**a**) N_2_ adsorption–desorption isotherms and (**b**) corresponding pore size distributions of the NUT-21 and NUT-21-TETA.

The porosity and specific surface area of the samples were evaluated by N_2_ adsorption–desorption isotherms at 77 K (Figure 5), and the related structural parameters were calculated, respectively (Table 1). The sorption curves of the NUT-21 and NUT-21-TETA were mainly type I isotherms with a fast N_2_ adsorption in a low relative pressure (*P/P*_0_) range, indicating the presence of micropores. The hysteresis loops shown in the isotherms of both the NUT-21 and NUT-21-TETA (Figure 5a) suggested the existence of mesoporous structures. These measurement results are consistent with the pore size distribution (Figure 5b). Compared with that of the counterpart NUT-21, the N_2_ uptake of the NUT-21-TETA decreased. The BET surface area of the NUT-21-TETA (665 m^2^/g) was lower than that of the NUT-21 (722 m^2^/g), which was attributed to the steric hindrance effect of the incorporated amino groups. As displayed in Figure 5b, the hierarchical pore structures were observed for both the NUT-21 and NUT-21-TETA, which provide good accessibility to the metal ion trap. This is because the broad mesopores benefit the transport of the metal ions, and the micropores facilitate the interaction between the chelating sites and the metal ions [17].

### 2.2. Metal Ion Adsorption Performance

Generally, the removal capacity of adsorbents with amine groups suffers from the pH disturbance because of the competitive adsorption between the heavy metal ions and H^+^ in the low pH range. The effect of pH value on the Pb^2+^ capture of the NUT-21-TETA was presented in Figure S2. At a low pH value, the amino functional groups get protonated, and the non-negligible electrostatic repulsive force restrains the approach of Pb^2+^ to the active sites of the NUT-21-TETA, thus resulting in an undesirable Pb^2+^ removal performance. The amino groups become non-protonated and the electrostatic repulsive force declines gradually with a decrease in the acidity of the solution. The free amino groups can act as surface-bound ligands to combine efficiently with the targeted Pb^2+^, being responsible for the improvement of removal capacity accordingly. The Pb^2+^ hydroxide precipitated when the pH of the aqueous solution rose to about 8. The following adsorption measurements were conducted at pH 6.1, except as otherwise noted.

**Figure 6:**
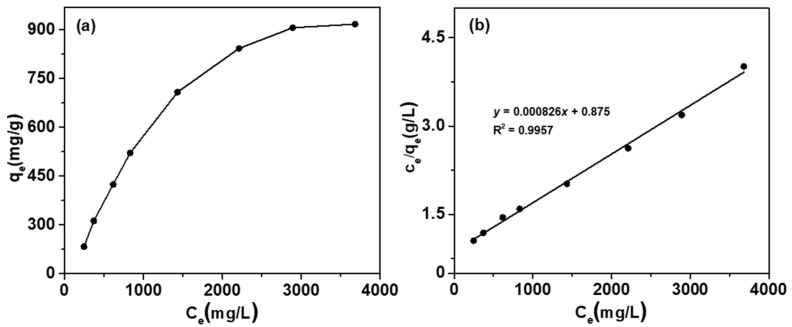
(**a**) Pb^2+^ adsorption isotherm by the NUT-21-TETA, (**b**) Linear regression by fitting the equilibrium adsorption data with Langmuir adsorption model. (m = 50 mg, V = 10 mL, T = 25 °C).

In order to examine the heavy metal ion adsorption performance of the NUT-21-TETA from the aqueous solution, the adsorption isotherm was collected at 25 °C. As displayed in Figure 6a, the equilibrium adsorption data of Pb^2+^ on the NUT-21-TETA demonstrated that the uptake increased significantly with the growing of the Pb^2+^ equilibrium concentration, while the adsorption isotherms tended to be even when the Pb^2+^ concentration was near 3000 mg/L. The NUT-21-TETA exhibited better Pb^2+^ capacity than its counterpart NUT-21 (Figure S3) because of the incorporation of the amino groups. The experimental data were fitted using the Langmuir approach, which yielded a high correlation coefficient (R^2^ > 0.99) (Figure 6b and Table S1). This excellent correlation of the measurement results with the Langmuir adsorption model indicated the monolayer adsorption behavior of Pb^2+^, and the chemical adsorption on the NUT-21-TETA. Surprisingly, the maximum Pb^2+^ uptake of the NUT-21-TETA (q_m_) is as high as 1211 mg/g, which is much superior to that of the non-functionalized sample of the NUT-21 (39 mg/g), as well as many adsorbents reported such as the ZIF-8 (1120 mg/g) [40], FGO(842 mg/g) [41], TBN-1 (730 mg/g) [42], TM-HPS (693 mg/g) [43], POP-NH_2_ (524 mg/g) [44], activated carbon (AC) (58 mg/g) [45], amino-functionalized MIL-101(Cr) (81 mg/g) [46], 4A zeolite (283 mg/g), zeolite 13X (541 mg/g) [47], and 732-CR resin (397 mg/g) [48] (Table S2). The promising Pb^2+^ adsorption performance achieved can be attributed to the fact that NUT-21-TETA possesses abundant porosity and high-density amino groups, acting as Pb^2+^-philic adsorption sites to improve the adsorption process.

**Figure 7:**
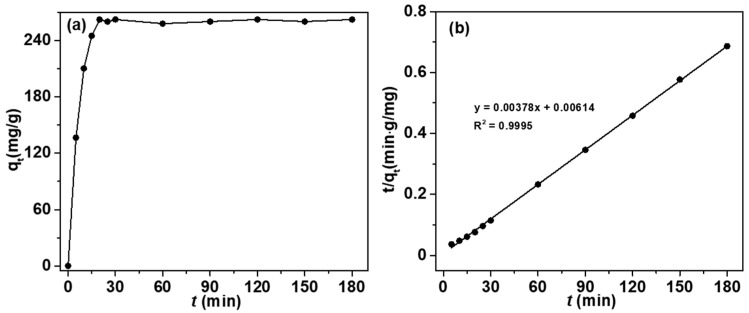
(**a**) Adsorption kinetic curves of the NUT-21-TETA for Pb^2+^ removal, (**b**) the pseudo-second-order kinetic plot for the adsorption (C_0_ = 500 mg/L, m = 100 mg, V = 200 mL, T = 25 °C).

In addition, NUT-21-TETA demonstrated a rapid adsorption rate and the Pb^2+^ uptake reached equilibrium in just 20 min (Figure 7a). The Pb^2+^ adsorption kinetics of NUT-21-TETA was investigated and the uptake data were fitted by a pseudo-second-order kinetic model (Figure 7b). An excellent fitting result with quite a high correlation coefficient (R^2^ > 0.999) was obtained (Table S3), indicating that the Pb^2+^ capture mechanism was chemisorption and the removal rate of Pb^2+^ depended on the availability of adsorption active sites [49].

From the practical application point of view, the recyclability of adsorbents is the most critical property. The effect of pH on the Pb^2+^ removal of the NUT-21-TETA (Figure S2) indicates that an excellent combination exists between the NUT-21-TETA and Pb^2+^ at pH 6.1, but less affinity at a low pH is indicated, meaning that the Pb^2+^ desorption of the NUT-21-TETA could be realized at a low pH. For instance, the NUT-21-TETA can be regenerated and reused for the Pb^2+^ trap by rinsing it with 0.1 M HCl. To examine the cycling stability of the NUT-21-TETA, an adsorption–desorption cycle was repeated five times, and the removal efficiency was still higher than 90%. As presented in Figure S4, no obvious decrease of Pb^2+^ uptake was observed during the adsorption–desorption recycles. These experimental results suggest that the NUT-21-TETA is promising for the efficient capture of Pb^2+^ from the aqueous solution.

Considering that most wastewater contains different heavy metal ions, we further examined the adsorption performance of the NUT-21-TETA on another five metal ions. As displayed in Figure S5, the removal efficiencies of the NUT-21-TETA on various metal ions were all higher than 90%, demonstrating a broad adsorption capability for different metal ions. For the divalent ions, in agreement with their weak acidic change order, the removal efficiencies were in the order of Ni^2+^ < Cu^2+^ < Zn^2+^ < Pb^2+^, while in the case of the trivalent metal ions Cr^3+^ and Fe^3+^, comparable removal efficiencies were observed because of their hard, acidic, cationic nature. To simulate a flow-through process of water purification, the column breakthrough curve measurement was conducted in a fixed bed. The binary metal ion mixtures Pb^2+^/Ni^2+^ (50/50, n/n) was used. As depicted in Figure S6, the Ni^2+^ was detected firstly in the outlet of the fixed bed, which was in accordance with the single-component test result, and this can also be attributed to the weak acidic change.

## 3. Conclusions

In summary, a two-step strategy was developed for the successful design and preparation of amine-enriched PPGs. The precursor polymer gel NUT-21 was synthesized by a simple Friedel–Crafts reaction, and then the amino groups, as hydrophilic adsorption sites, were efficiently incorporated via post-synthetic functionalization. The target adsorbent, NUT-21-TETA, was used for the Pb^2+^ capture from aqueous solution, reaching an extremely high Pb^2+^ uptake of 1211 mg/g. The efficient Pb^2+^ removal was attributed to the synergetic effect of the developed hierarchical pore structure and the high density of amino groups with a high affinity for Pb^2+^. Moreover, the NUT-21-TETA can be completely regenerated and reused. The outstanding adsorption capacity, excellent reusability, and low-cost preparation endows the NUT-21-TETA in this study with a strong potential for the removal of toxic heavy metal ions from wastewater. Such a facile synthetic protocol might be further extended to the development of other high-performance functional materials.

## 4. Materials and Methods

### 4.1. Materials and Chemicals

Mesitylene (C_9_H_12_), 1,4-bis(Chloromethyl)-Benzen (C_8_H_8_Cl_2_), iron(III) chloride (FeCl_3_), 1,2-dichloroethane (C_2_H_4_Cl_2_), methanol (CH_3_OH), N-bromosuccinimide (C_4_H_4_BrNO_2_), anhydrous carbon tetrachloride (CCl_4_), N, N-tetrahydrofuran (C_4_H_8_O), dimethylformamide (C_3_H_7_NO), benzoyl peroxide (C_14_H_10_O_4_), triethylenetetramine (C_6_H_18_N_4_), and acetone (C_3_H_6_O) were purchased from Shanghai Titan Technology Co., Ltd., Shanghai, China. All chemicals purchased were used as received with no further treatment.

### 4.2. Synthesis of Materials

**Scheme 1:**
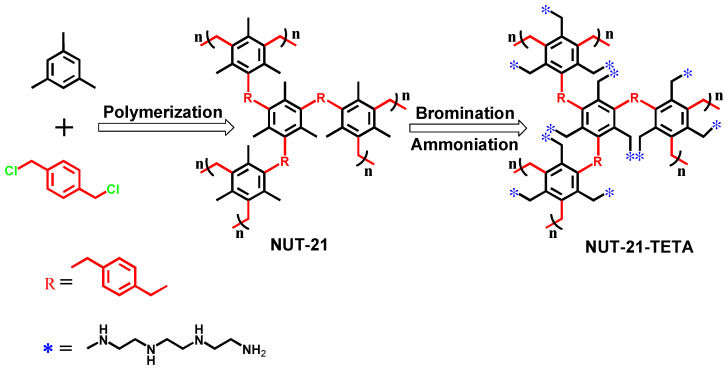
Synthetic scheme of NUT-21-TETA formed by Friedel–Crafts alkylation and post-synthetic functionalization.

The preparation process of the NUT-21-TETA followed a two-step strategy, as shown in Scheme 1. Firstly, the non-functionalized support NUT-21 was fabricated via a Friedel–Crafts alkylation reaction of mesitylene and 1,4-bis(Chloromethyl)-Benzen. In a typical operation, a closed vial is charged with mesitylene (50 mmol), 1,4-bis(Chloromethyl)-Benzen (150 mmol), and DCE (250 mL), and then the resultant mixtures were stirred for 1 h under the N_2_ atmosphere. Anhydrous FeCl_3_ (150 mmol) was subsequently added, after which the solution was heated and kept at 62 °C for 36 h to acquire the umber powder. When cooling to room temperature, the reaction mixtures were filtered and then purged using 2 M HCl solution, distilled water, DCE, and methanol, successively. Further washing of the precipitate with acetone was performed with a Soxhlet extractor for 2 h to wash away the unreacted reactants and the catalyst completely. The sample was treated under vacuum at 80 °C for 12 h to acquire the brown powder, denoted as NUT-21.

The bromomethylation of the NUT-21 was carried out to obtain the intermediate NUT-21-CH_2_Br. NUT-21 (1.5 g), N-bromosuccinimide (3.0 g), and benzoyl peroxide (1.0 g) were mixed in anhydrous CCl_4_ (250 mL) and then refluxed for 36 h at 80 °C. Then, cooling to room temperature, the mixtures were filtered and washed using DMF, methanol, deionized water, and acetone, successively, and dried under vacuum for 24 h at 80 °C to yield the NUT-21-CH_2_Br.

Amino groups were introduced via the post-modification method. The as-prepared NUT-21-CH_2_Br (0.1 g) was dispersed in 25.0 mL tetrahydrofuran by stirring for 2 h to form a uniform mixture, and then 0.3 g NaOH and 15.0 mL TETA were added into the mixture. The reflux was performed at 62 °C for 24 h with vigorous stirring. When the reaction finished, the above mixture was cooled to 25 °C and then purged fully using the mixture of ethanol/water (50 mL/50 mL) and dried under vacuum for 24 h at 80 °C to obtain the target NUT-21-TETA.

### 4.3. Structural Characterization

Fourier transform infrared (FT-IR) spectra of the samples were collected by a Thermo Scientific Nicolet 380 FTIR spectrometer using the KBr pellet technique. The X-ray photoelectron spectroscopy (XPS) measurement was conducted on the Physical Electronic PHI-550 spectrometer. The X-ray diffraction (XRD) patterns of the materials were conducted on an Empyrean diffractometer using Cu K_α_ radiation (λ = 1.5 Å) in the 2*θ* ranging from 5° to 60° at 100 kV and 40 mA. The morphology and particle sizes of the samples were observed by field-emission scanning electron microscopy (Hitachi S4800, running at 2 kV) and a high-resolution transmission electron microscope (HRTEM, JEM-200CX at 200 kV). The solid state ^13^C CP/MAS NMR spectra were performed with a Bruker AVANCE 400 spectrometer. Elemental analysis for the C, H, and N proportion of the samples was carried out with the elemental analyzer (UNICUBE). Thermogravimetric analyses (TGA) were performed with a thermobalance (STA-499C, NETZSCH). The surface area and pore size were analyzed with N_2_ adsorption–desorption isotherms at 77 K on Micromeritics ASAP-3020. The Brunauer–Emmett-Teller (BET) model was used to calculate the specific surface areas within a relative pressure (*P/P*_0_) region of 0.05 to 0.15. The total pore volumes were determined with adsorption at the *P/P*_0_ of 0.95. Pore size distribution (PSD) was estimated with the Barrett–Joyner–Halenda approach. The metal ion concentration of the model solution was analyzed with inductively coupled plasma–mass spectrometry (ICP, Agilent 7700CE).

### 4.4. Batch Adsorption Experiment

The adsorption capacities of different metal ions were investigated in static batch experiments under various beginning concentrations of Pb^2+^ and other metal ions including Ni^2+^, Cu^2+^, Zn^2+^, Cr^3+^, and Fe^3+^. The pH of aqueous solutions was regulated by either 0.1 M sodium hydroxide or 0.1 M hydrochloric acid solution. The capture amount (q_e_) and removal efficiency (E%) were calculated as follows [17]:

(1) $$q_{e}=\frac{(C_{0}-C_{e})\times V}{m}$$

(2) $$E\%=\frac{\left(C_{0}-C_{e}\right)}{C_{0}}\times 100\%$$

where C_0_ denotes the beginning concentration (mg/L), C_e_ means the equilibrium concentration of metal ions in the model solution (mg/L), V refers to the volume of the adsorption solution (mL), and m denotes the amount of the dried adsorbent used (mg).

#### 4.4.1. Adsorption Thermodynamics Test

To investigate the Pb^2+^ adsorption thermodynamics, 50 mg NUT-21-TETA was placed in the glass bottle charged with 10 mL of Pb^2+^ solution. The beginning Pb^2+^ content was from 100 mg/L to 4000 mg/L. The bottle was kept at 25 °C for 12 h in a constant temperature furnace. After that, the mixtures were centrifuged and filtered, and then the filtrates were tested using ICP to calculate the Pb^2+^ uptake. The Langmuir adsorption equation, Equation (3), was employed to evaluate the adsorption thermodynamics [17].

(3) $$E\%=\frac{\left(C_{0}-C_{e}\right)}{C_{0}}\times 100\%$$

In Equation (3), C_e_ (mg/L) means the removal equilibrium concentration, q_e_ (mg/g) and q_m_ (mg/g) represent the equilibrium capture amount and the highest uptake of the heavy metal ion, respectively. K_L_ (L/mg) denotes the Langmuir constant.

#### 4.4.2. Adsorption Kinetics Test

To examine the Pb^2+^ adsorption kinetics, 0.1 g NUT-21-TETA was added into a bottle with 200 mL Pb^2+^ solution containing the starting Pb^2+^ content of 500 mg/L. The mixtures were kept at 25 °C, and the Pb^2+^ concentration was examined at different time intervals. The pseudo-second-order kinetic equation, Equation (4), was used to investigate the adsorption kinetics [17,50,51].

(4) $$\frac{t}{q_{t}}=\frac{1}{K_{2}q_{e}{}^{2}}+\frac{1}{q_{e}}t$$

where t means the removal time, q_t_ (mg/g) and q_e_ (mg/g) are the uptakes of heavy metal ion at time t and equilibrium, respectively; K_2_ means the rate constant of the pseudo-second-order kinetics.

#### 4.4.3. Various Metal Ions Adsorption Test

To explore the adsorption capacity of the NUT-21-TETA on different metal ions such as Pb^2+^, Ni^2+^, Cu^2+^, Zn^2+^, Fe^3+^, and Cr^3+^, 50 mg NUT-21-TETA was placed in a bottle containing 10 mL heavy metal ion solution with a starting content of 0.5 mmol/L. The mixtures were stirred and kept at 25 °C for 12 h.

#### 4.4.4. Recyclability Test

The reusability test of the NUT-21-TETA was evaluated by a desorption and regeneration process. After each Pb^2+^ adsorption, the NUT-21-TETA was separated by the centrifugation and purged completely with 0.1 M EDTA-2Na solution, after which it was dried at 85 °C for 6 h. The regenerated sample was re-employed for Pb^2+^ uptake 5 times.

#### 4.4.5. Breakthrough Experiment

The breakthrough test was conducted to investigate the heavy metal ion removal ability of the NUT-21-TETA, which was fulfilled in a fixed bed (a glass tube column with an internal diameter of 10 mm packed with 0.5 g dry NUT-21-TETA). The binary metal ion mixtures, Pb^2+^/Ni^2+^ (50/50, n/n), were employed for dynamic breakthrough measurement. The metal ion concentration of the model solution in the column outlet was analyzed with the ICP.

## Figures and Tables

**Table 1:** Textural parameters and elemental analysis data of the resultant samples.

Sample	Yield (%, wt.)	*S*_BET_ (m^2^/g)	*V*_tot_ (cm^3^/g)	EA (%, wt.)		
				N	C	H
NUT-21	87.3	722	1.37	0	85.45	4.55
NUT-21-TETA	79.8	665	1.05	9.06	69.36	6.91

## Data Availability

Not applicable.

## Supplementary Materials

The following supporting information can be downloaded at: https://www.mdpi.com/article/10.3390/gels9040297/s1, Figure S1: XRD patterns of the samples; Figure S2: Effect of solution pH on Pb^2+^ adsorption; Figure S3: Pb^2+^ adsorption isotherm by the NUT-21; Figure S4: Cycling removal efficiency of Pb^2+^ on NUT-21-TETA (C_0_ = 100 mg/L, m = 50 mg, V = 10 mL, T = 25 °C); Figure S5: Adsorption performance of NUT-21-TETA for different metal ions (C_0_ = 100 mg/L, m = 50 mg, V = 10 mL, T = 25 °C); Figure S6: (a) Breakthrough test set-up, (b) breakthrough curves of the model aqueous solution containing 500 mg/L Pb^2+^/Ni^2+^ (50/50, n/n) over NUT-21-TETA; Table S1: Langmuir model parameters for the Pb^2+^ uptake on NUT-21-TETA and NUT-21; Table S2: Comparison of the adsorption performance for different adsorbents; Table S3: Pseudo-second-order kinetic model parameters for the Pb^2+^ uptake on NUT-21-TETA. References [52,53,54,55,56,57,58,59,60,61] are cited in the supplementary materials.
