# Supplementary material for: Highly Efficient Capture of Heavy Metal Ions on Amine-Functionalized Porous Polymer Gels
Source: Gels. 2023 Apr 2;9(4):297. doi: 10.3390/gels9040297 (PMC10137378; doi:10.3390/gels9040297)
Supplement: Supplementary file 1 [file gels-09-00297-s001.zip › gels-2286693-supplementary.pdf]

Supplementary Material

Highly Efficient Capture of Heavy Metal Ions on Amine-functionalized Porous Polymer Gels

Xue He †, Jumu Xia †, Jieli He, Kezhen Qi, Anzhong Peng * and Yong Liu *

College of Pharmacy, Dali University, Dali 671003, China
* Correspondence: penganzhong@dali.edu.cn (A.P); liuy@dali.edu.cn (Y.L)
† These authors contributed equally to this work.

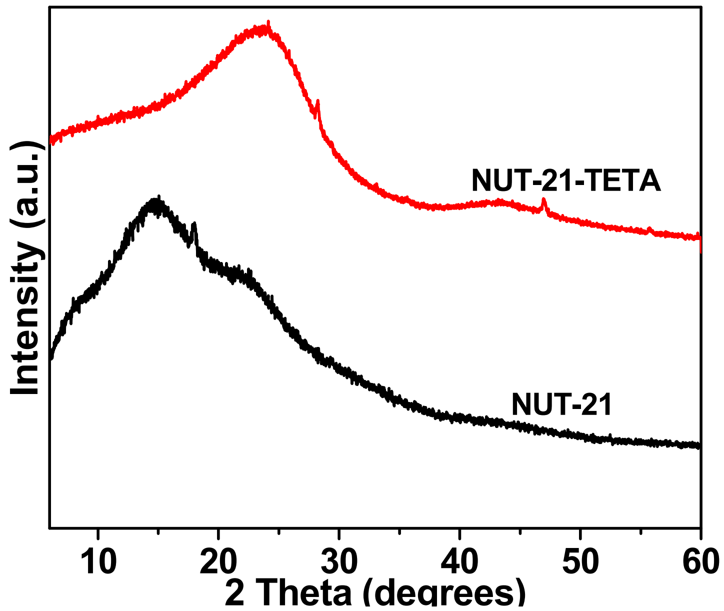

Figure S1. XRD patterns of the samples

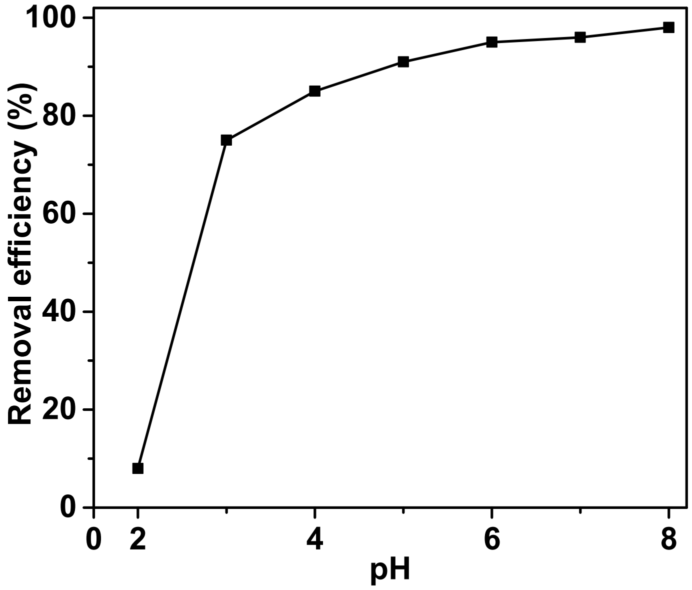

Figure S2. Effect of solution pH on Pb^{2+} adsorption.

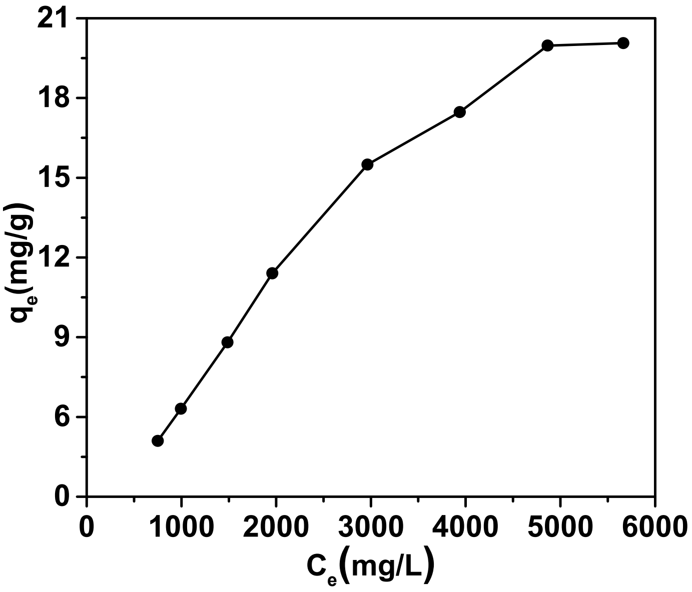

Figure S3. Pb^{2+} adsorption isotherm by the NUT-21.

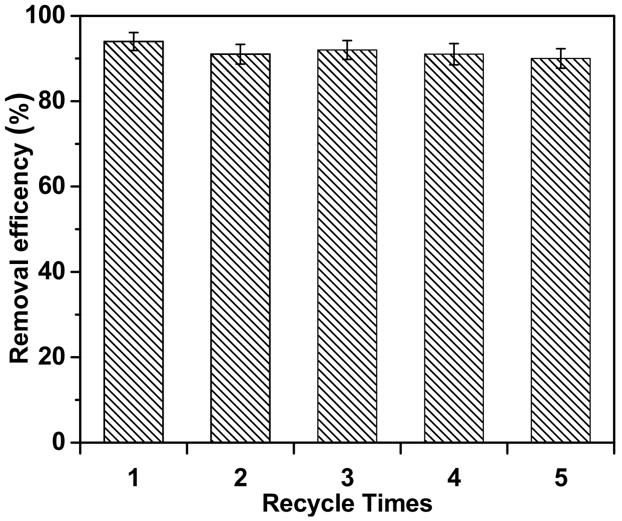

Figure S4. Cycling removal efficiency of Pb^{2+} on NUT-21-TETA (C_0 = 100 mg/L, m = 50 mg, V = 10 mL, T = 25 °C).

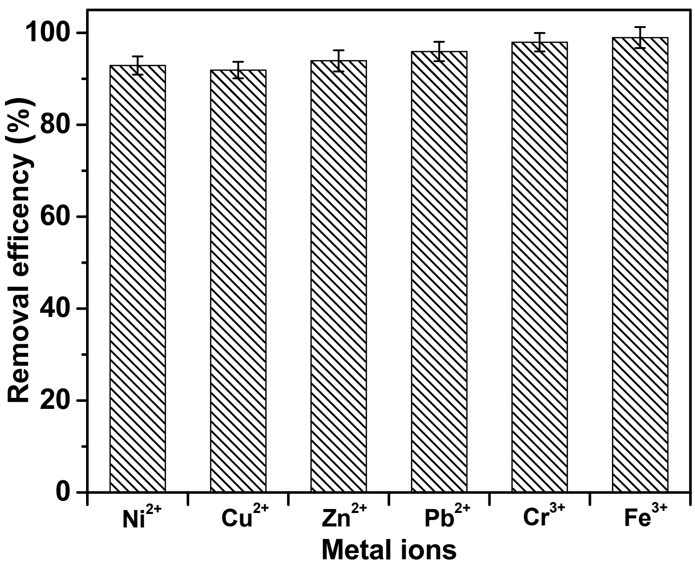

Figure S5. Adsorption performance of NUT-21-TETA for different metal ions (C_0 = 0.5 mmol/L, m = 50 mg, V = 10 mL, T = 25 °C).

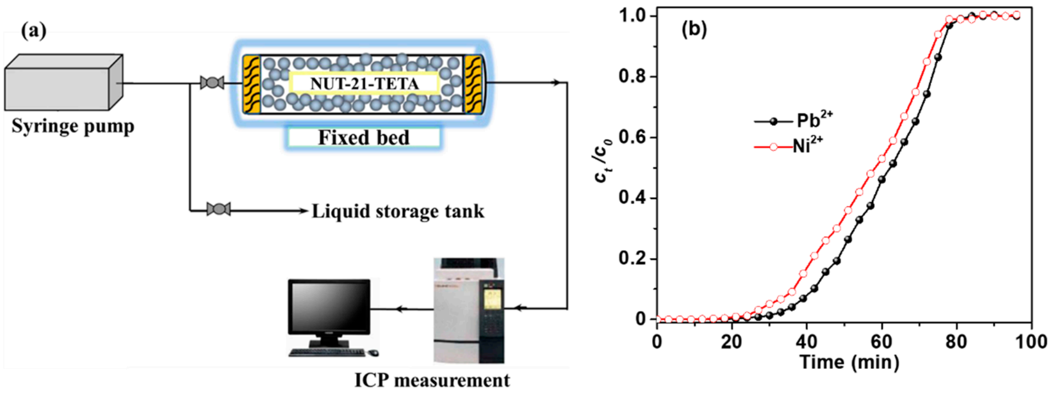

Figure S6. (a) Breakthrough test set-up, (b) breakthrough curves of the model aqueous solution containing 2.5 mmol/L Pb^{2+}/Ni^{2+} (50/50, n/n) over NUT-21-TETA.

Table S1 Langmuir model parameters for the Pb^{2+} uptake on NUT-21-TETA and NUT-21.

Adsorbents	Metal ion	Langmuir equation	Langmuir model		
			q_{max} (mg/g)	K_L(L/mg)	R^2
NUT-21-TETA	Pb^{2+}	$C_e/q_e = 0.000826C_e + 0.875$	1211	0.0009437	0.9957
NUT-21	Pb^{2+}	$C_e/q_e = 0.02546C_e + 26.52488$	39	0.0002026	0.9723

Table S2 Comparison of the adsorption performance for different adsorbents

Adsorbents	q_{max} (mg/g)	Ref.
AC	58	1
CTAB mesoporous silica	59	2
amino-functionalized MIL-101(Cr)	81	3
4A zeolite	283	4
Zeolite 13X	541	4
EDTA-mGO	508	5
TETA-g-PAM/O Verm	219	6
SBA-15	103	7
Fe_3O_4@SiO_2-EDTA nanocomposites	115	8
PS-TETA resin	149	9
732-CR resin	397	10
POP-NH_2	524	11
Amidoxime-functionalized SiO_2	284	12
MoS_2 microrods/C	303	13
Co-Fe_2O_3	136	14
Chitosan modified with Glu-IL-CS	149	15
TM-HPS	693	16
$CoFe_2O_4$NPs	602	17
TBN-1	730	18
FGO	842	19
ZIF-8	1120	20
NUT-21	39	This work
NUT-21-TETA	1211	**This work**

Table S3 Pseudo-second-order kinetic model parameters for the Pb^{2+} uptake on NUT-21-TETA.

**Adsorbent**	**Metal ion**	**Pseudo-second-order kinetic equation**	**Pseudo-second-order**	
			**K_2(g/mg/min)**	**R^2**
NUT-21-TETA	Pb^{2+}	$t/q_t = 0.00378t + 0.00614$	0.0023	0.9995

References

[1] F. Alguacil, L. Alcaraz, I. García-Díaz, F. López, 2018. Removal of Pb^{2+} in wastewater via adsorption onto an activated carbon produced from winemaking waste. Metals 8, 1-15.

[2] N. Wang, X. Xu, H. Li, J. Zhai, L. Yuan, K. Zhang, H. Yu, 2016. Preparation and application of a xanthate-modified thiourea chitosan sponge for the removal of Pb(II) from aqueous solutions. Industrial & Engineering Chemistry Research 55, 4960-4968.

[3] X. Luo, L. Ding, J. Luo, 2015. Adsorptive removal of Pb(II) ions from aqueous samples with amino-functionalization of Metal–Organic Frameworks MIL-101(Cr). Journal of Chemical and Engineering Data 60, 1732-1743.

[4] M. Chen, S. Nong, Y. Zhao, M.S. Riaz, Y. Xiao, M.S. Molokeev, F. Huang, 2020. Renewable P-type zeolite for superior absorption of heavy metals: Isotherms, kinetics, and mechanism. Science of the Total Environment 726, 1-32.

[5] L. Cui, Y. Wang, L. Gao, L. Hu, L. Yan, Q. Wei, B. Du, 2015. EDTA functionalized magnetic graphene oxide for removal of Pb(II), Hg(II) and Cu(II) in water treatment: Adsorption mechanism and separation property. Chemical Engineering Journal 281, 1-10.

[6] S. Gu, L. Wang, X. Mao, L. Yang, C. Wang, 2018. Selective adsorption of Pb(II) from aqueous solution by triethylenetetramine-grafted polyacrylamide/vermiculite. Materials (Basel) 11.

[7] R. He, W. Li, D. Deng, W. Chen, H. Li, C. Wei, Y. Tang, 2015. Efficient removal of lead from highly acidic wastewater by periodic ion imprinted mesoporous SBA-15 organosilica combining metal coordination and co-condensation. Journal of Materials Chemistry A 3, 9789-9798.

[8] Y. Liu, R. Fu, Y. Sun, X. Zhou, S.A. Baig, X. Xu, 2016. Multifunctional nanocomposites $Fe_3O_4@SiO_2$ - EDTA for Pb(II) and Cu(II) removal from aqueous solutions. Applied Surface Science 369, 267-276.

[9] C. Xiong, C. Yao, 2009. Synthesis, characterization and application of triethylenetetramine modified polystyrene resin in removal of mercury, cadmium and lead from aqueous solutions. Chemical Engineering Journal 155, 844-850.

[10] H. Guo, Y. Ren, X. Sun, Y. Xu, X. Li, T. Zhang, J. Kang, D. Liu, 2013. Removal of Pb^{2+} from aqueous solutions by a high-efficiency resin. Applied Surface Science 283, 660-667.

[11] Y. He, Q. Liu, J. Hu, C. Zhao, C. Peng, Q. Yang, H. Wang, H. Liu, 2017. Efficient removal of Pb(II) by amine functionalized porous organic polymer through post-synthetic modification. Separation and Purification Technology 180, 142-148.

[12] Y. Xie, J. Wang, M. Wang, X. Ge, 2015. Fabrication of fibrous amidoxime-functionalized mesoporous silica microsphere and its selectively adsorption property for Pb(2+) in aqueous solution. Journal of Hazardous materials 297, 66-73.

[13] N. Kumar, E. Fosso-Kankeu, S.S. Ray, 2019. Achieving controllable MoS_2 nanostructures with increased interlayer spacing for efficient removal of Pb(II) from aquatic systems. ACS Applied Materials & Interfaces 11, 19141-19155.

[14] W. Chen, Z. Lu, B. Xiao, P. Gu, W. Yao, J. Xing, A.M. Asiri, K.A. Alamry, X. Wang, S. Wang, 2019. Enhanced removal of lead ions from aqueous solution by iron oxide nanomaterials with cobalt and nickel doping. Journal of Cleaner Production 211, 1250-1258.

[15] H. Ren, B. Li, M. Neckenig, D. Wu, Y. Li, Y. Ma, X. Li, N. Zhang, 2019. Efficient lead ion removal from water by a novel chitosan gel-based sorbent modified with glutamic acid ionic liquid. Carbohydrate Polymers 207, 737-746.

[16] Z. Yang, X. Huang, X. Yao, H. Ji, 2018. Thiourea modified hyper-crosslinked polystyrene resin for heavy metal ions removal from aqueous solutions. Journal of Applied Polymer Science 135, 1-13.

[17] E. Nazarzadeh Zare, A. Mudhoo, M. Ali Khan, M. Otero, Z.M.A. Bundhoo, M. Patel, A. Srivastava, C. Navarathna, T. Mlsna, D. Mohan, C.U. Pittman, Jr., P. Makvandi, M. Sillanpaa, 2021. Smart adsorbents for aquatic environmental remediation. Small 17, 1-31.

[18] H. Shan, S. Li, Z. Yang, X. Zhang, Y. Zhuang, Q. Zhu, D. Cai, P. Qin, J. Baeyens, 2021. Triazine-based N-rich porous covalent organic polymer for the effective detection and removal of Hg (II) from an aqueous solution. Chemical Engineering Journal 426, 1-10.

[19] G. Zhao, X. Ren, X. Gao, X. Tan, J. Li, C. Chen, Y. Huang, X. Wang, 2011. Removal of Pb(II) ions from aqueous solutions on few-layered graphene oxide nanosheets. Dalton Trans 40, 10945-10952.

[20] Y. Huang, X. Zeng, L. Guo, J. Lan, L. Zhang, D. Cao, 2018. Heavy metal ion removal of wastewater by zeolite-imidazolate frameworks. Separation and Purification Technology 194, 462-469.
